# Protection effect of taurine on nitrosative stress in the mice brain with chronic exposure to arsenic

**DOI:** 10.1186/1423-0127-17-S1-S7

**Published:** 2010-08-24

**Authors:** Ning Ma, Mikio Sasoh, Shosuke Kawanishi, Hiromichi Sugiura, Fengyuan Piao

**Affiliations:** 1 Faculty of Health Science and Institute of Traditional Chinese Medicine, Suzuka University of Medical Science, Suzuka, Mie 510-0293, Japan; 2 Department of Ophthalmology, Mie University Graduate School of Medicine, Tsu 514-0001, Mie, Japan; 3 Department of Hygiene, Dalian Medical University, Dalian 116027, China

## Abstract

**Background:**

Arsenic exposure induces overproduction of reactive nitrogen species (RNS) in brain tissue and results in nucleic acid damage to the nerve cells. The 8-nitroguanine is one of the major products formed by the reaction of guanine, and ONOO^-^, and has been used as a popular biomarker of nucleic acid damage due to RNS attacking. In the present study, we examined whether the administration of taurine can protect against nucleic acid damage of brain neurons by arsenic-induced RNS.

**Materials and methods:**

Sixty mice (30 male and 30 female) weighing 19.5 ± 1.5 g were divided into 3 groups: (1) control group, (2) experimental group that received arsenic (As_2_O_3_), and (3) antagonistic group that received taurine with arsenic. Arsenic was administered for 60 days. 8-Nitroguanine expressions in brain neurons of mice were examined by the immunohistochemical method. Histopathological changes in brain tissues of mice were observed under light microscope and the immunohistochemistry method was used to investigate 8-nitroguanine expressions in cerebrum and cerebellum of mice.

**Results:**

In the control group, no abnormal histopathological changes were observed in brain tissue of the mice. In brain tissue of the mice exposed to arsenic, histopathological results showed swells, evident vacuolar degeneration in cytoplasm, karyorrhexis and karyolysis. Relatively light pathological changes were observed in brain of the mice co-administered arsenic and taurine. Little or no expression of 8-nitroguanine in brain tissue was observed in controls. However, intensive expression of 8-nitroguanine was found in brain tissue of mice exposed to arsenic and it was mainly distributed in nucleus neighbouring the nuclear membrane, but a little in cytoplasm. A weak expression of 8-nitroguanine was observed in brain cells of mice co-administered arsenic and taurine.

**Conclusions:**

The brain neurons may be the major target cells of arsenic neurotoxicity. Co-administration of arsenic and taurine can alleviate DNA damage of brain neurons caused by arsenic through the RNS signal pathway.

## Background

Arsenic is a naturally occurring element that is ubiquitously present in the environment. High concentration of naturally occurring arsenic in drinking water is a major health problem in different parts of the world. Arsenic is an environmental contaminant found naturally in ground water. Drinking water contamination by arsenic remains a major public health problem [1]. Acute and chronic arsenic exposure via drinking water has been reported in many countries of the world. There are sufficient epidemiological evidences revealing a causal association between arsenic exposure and human disease. Arsenic is also a neurotoxical substance. Drinking water containing arsenic exceeding 10 μg/L is harmful to the body [2]. Arsenic contamination also results from industrial and agricultural uses [3]. The adverse effect of acute and sub-chronic exposures to arsenic on the nervous system has been receiving more and more attention. Epidemiological research demonstrated that exposure to arsenic results in impaired learning and concentration for studying, and deteriorated pattern memory and attention deficits in humans [4,5]. It was shown in animal experiments that arsenic could pass through the blood-brain barrier and invade the brain parenchyma, and there was a noticeable correlation between the dose of arsenic exposure and its concentration in the brain of guinea pigs and rats. Arsenic exposure renders the brain tissue vulnerable to radical attack resulting in abnormal apoptosis of neural cells. However, the mechanism of arsenic induced neurotoxicity is unclear to date [6]. It is known that arsenic exposure induces overproduction of reactive oxygen species (ROS) and reactive nitrogen species (RNS) in the body and results in nucleic acid damage to the nerve cells [7], which is one of mechanisms of arsenic toxicity. Therefore, it indicated that adverse effect of arsenic on bio-macromolecule maybe avoided or mitigated by intervention of antioxidants.

8-Nitroguanine is a mutagenic nitrosative DNA lesion caused by reactive nitrogen and oxygen species, and now has been used as a potential biomarker of inflammation-related cancers [8]. In the present study, 8-nitroguanine was used as a biomarker of nucleic acid damage [9]. We examined by the immunohistochemical method the interfering effects of taurine as antioxidants on nucleic acid damage of mice brain tissue exposed to arsenic to provide experimental evidences for prevention and therapy for the arsenic induced brain damage.

## Materials and methods

### Animal

Sixty mice (Slc/ICR, 30 male and 30 female) weighing 19.5 ± 1.5 g were purchased from Japan SLC (Shizuoka, Japan) and maintained under specific pathogen free conditions at the Institute for Laboratory Animals of Suzuka University of Medical Science. They were caged under a 12 h dark-light cycle in standard conditions of temperature (18–22 C) and humidity (50 %). The animals were maintained on a standard diet and water* ad libitum*. The animal experiment was performed in accordance with the Animal Guideline of Suzuka University of Medical Science and in agreement with the Ethical Committee of Suzuka University of Medical Science. Mice were 6 weeks old at the beginning of each experiment. All the mice were sex matched and weighed before the beginning of the experiment and pooled together. From this pool, the mice were randomly assigned to three different groups of twenty each. Group 1 received drinking water alone (as controls). Group 2 received 4 mg/L arsenic trioxide (as experimental group). Group 3 received both of 4 mg/L arsenic trioxide and 150 mg/kg taurine (as antagonistic apply group). Arsenic trioxide was given through drinking water for 60 days and taurine was affused to the stomach twice a week. After the last administration, mice were deeply anesthetized with an intraperitoneal injection of sodium pentobarbital (30 mg/kg) and were perfused transcardially with phosphate-buffered saline (PBS; 0.01 M sodium phosphate buffer, pH 7.4, 150 mM NaCl) for 1–2 min., followed by a fixative, containing 4% paraformaldehyde, in PBS, pH 7.0. After the perfusion, the brains were removed, and allowed to stand in the same fixative for 8 hours at 4°C, then rinsed several times with PBS, and kept at 4°C in PBS with 0.2% sodium azide until use.

### Histopathological examination

The formalin-fixed brain tissues were embedded in paraffin, sliced at 6 μm thickness, mounted on silanized slides, and subjected to hematoxylin and eosin staining according to the routine histopathological methods. Histopathological changes were observed under an Olympus Type BX51 microscopy.

### Immunohistochemical study

We produced specific anti-8-nitroguanine antibody using 8-nitroguanine-RSA-conjugates. Rabbit polyclonal anti-8-nitroguanine antibody without cross reaction was produced as described previously [10,11]. The antibody was purified using an 8-nitroguanine-conjugate column. Specificity of the purified antibody was examined by a dot immunobinding assay and absorption test [10]. Purified antibody gave a strong immunostaining only on the spot of 8-nitroguanine conjugate. The immunoreactivity disappeared only when the antibody was pre-incubated with 8-nitroguanine. In contrast, immunoreactivity with 8-nitroguanine conjugate did not disappear when the antibody was pre-incubated with 3-nitrotyrosine, guanosine, 8-oxodG, deoxyguanosine, 8-bromoguanosine, and xanthosine. This purified antibody was presented the immunoreactivity with free or DNA-associated 8-nitroguanine both. 8-Nitroguanine immunoreactivities in the mouse brain sections were assessed by a peroxidase anti-peroxidase (PAP) method study as described previously [12]. Briefly, paraffin sections (6-µm thickness) were incubated with rabbit polyclonal anti-8-nitroguanine antibody (2 µg/ml) overnight at room temperature. Then, the sections were incubated for 3 hours with goat antibody against rabbit IgG (1:200), and were followed by peroxidase anti-peroxidase complex (1:200). The sections that had been treated with first and second antibodies were incubated for 10 min at RT with 3,3’-diaminobentidine tetrahydrochloride (Dojindo Chemical Institute; Kumamoto, Japan) as chromogen, which had been freshly prepared as a solution of 20 mg in 100 ml PBS that contained 0.01% H_2_0_2_.

### Statistical analysis

The experimental results are expressed as the mean ± S.E.M. for twenty mice in each group, and the significant difference was analyzed by Student’s t-test. A *P* value of < 0.05 was considered to be statistically significant. We analyzed the correlations of the number of 8-nitroguanine immunopositive cell in each experiment group.

## Results

### Quantitative analysis of the experimental animals

**Figure 1:**
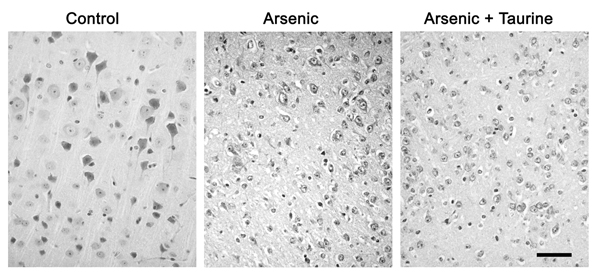
**Histopathological changes in brain tissue of mice from each group.** Pathological findings in the neuron of mice exposed to arsenic are observed. Axonal lose, cell shrinkage, evident vacuolar degeneration in cytoplasm and nucleus, karyorrhexis, and karyolysis are apparent. Relatively slight pathological changes are observed in cells from mice administered both arsenic and taurine. No abnormal histological changes in the brain tissues are observed in the control group. Bar = 50 µm.

All the 60 mice were involved in the analysis of results without deletion. Alteration of histopathological study in the brain tissues of mice administrated with arsenic or arsenic/taurine (Fig. 1).

In the hematoxylin and eosin stained brain sections, well stained neurons with a distinct nucleus and nucleolus along with well-stained Nissl substance in the cytoplasm formed a prominent feature of the cerebra and cerebella in the control group. The neuronal processes as well as the neuropile appeared well formed in the control group. Microscopic observations of arsenic administrated group brain sections revealed structural degeneration of neuron. The cerebral neuron cells of the mice exposed to arsenic showed cell swelling and evident vacuolar degeneration in cytoplasm and nucleus, karyorrhexis and karyolysis. Relatively light pathological changes were showed in the nervous cells of the mice administered both of arsenic and taurine.

### Formation of 8-nitroguanine in brain tissue of mice

**Figure 2:**
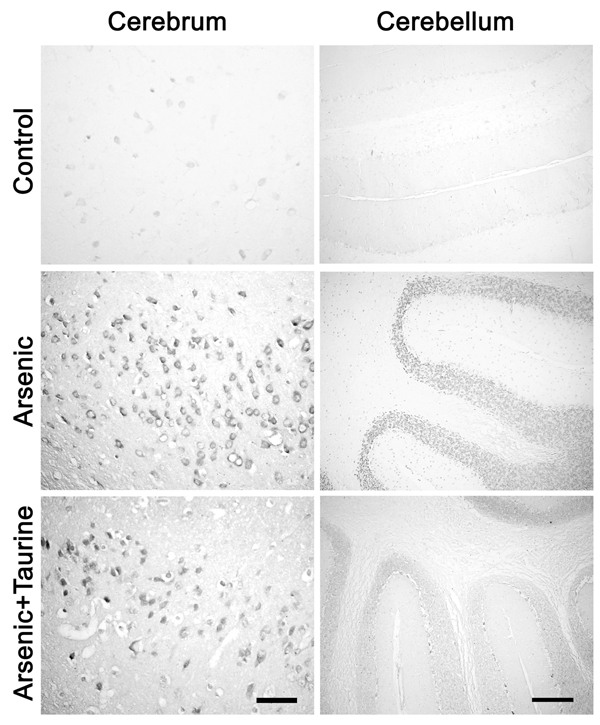
**8-Nitroguanine immunoreactivity in the neuron of cerebrum and cerebellum of mice from each group.** Little or no expression of 8-nitroguanine in brain tissue was observed in control group. Intensive expression of 8-nitroguanine was found in neuron cells in the cerebral cortex exposed to arsenic. Significantly elevated 8-nitroguanine immunoreactivity is observed in the granular cells of cerebellum of mice exposed to arsenic; 8-nitroguanine expression was primarily distributed in the nucleus. The density and number of 8-nitroguanine immunopositive cells were decreased in the cerebellum of mice administered both arsenic and taurine. Little or no 8-nitroguanine expression was observed in the cerebellum of control group. Bar: Cerebrum = 100 µm. Cerebellum = 200 µm.

8-nitroguanine immunoreactivity in the brain tissue of mice is shown in Fig. 2. Little or no expression of 8-nitroguanine in brain tissue was observed in control group (Fig. 2, Control). However, intensive expression of 8-nitroguanine was found in brain cells (Fig. 2, Arsenic) of mice exposed to arsenic. There was a tendency that 8-nitroguanine was localized in the neuron cells, whereas it was primarily distributed in the nucleus neighboring the nuclei membrane. The density and number of immunopositive cells were decreased in brain of mice administered both arsenic and taurine. Weak expression of 8-nitroguanine immunoreactivity was observed in neuron cells of mice that were administered both arsenic and taurine (Fig. 2, Arsenic+Taurine). In the cerebella, intensive expression of 8-nitroguanine was found in the Purkinje’s cells and granule cells. Relatively weak expression of 8-nitroguanine immunopositive cells was observed in granule cells of the cerebella that were administered both of arsenic and taurine.

### Quantitative analysis of the formation of 8-nitroguanine in the mice cerebrum

**Figure 3:**
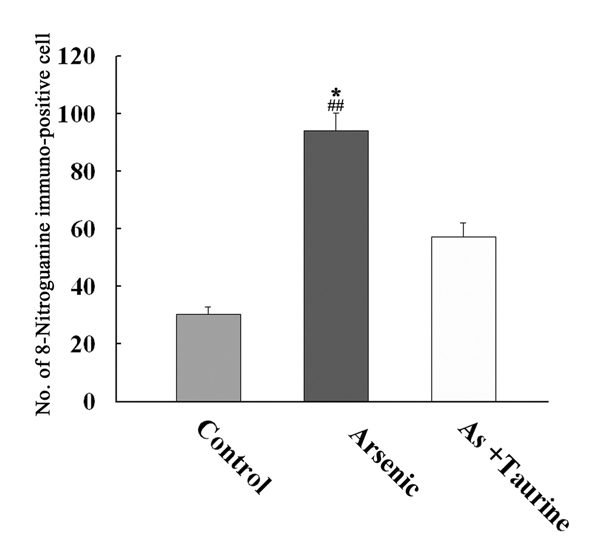
**Effect of taurine on the formation of 8-nitroguanine in the cerebral cortex of mice brain exposed to arsenic.** 8-Nitroguanine immunopositive cells in the cortex of mice brain in the control, arsenic and arsenic/taurine. Each microscopic scoring damage value represents the mean ± S.E.M of 10 microscopic fields in each animal. *P < 0.01 compared with the control group, and ^##^*P* < 0.01 compared with the arsenic+taurine group.

We counted the number of immunopositive neuron cells in the arsenic administrated group, arsenic/taurine administrated group and control group that exhibited immunoreactivity of 8-nitroguanine. Quantitative analysis of anti 8-nitroguanine staining neuron cells showed that the average immunopositive cells in brain tissue were significantly higher in the group exposed to arsenic than those in the other groups (*p* <0.01) as shown in Fig. 3.

## Discussion

Although many researches have demonstrated that arsenic is a neurotoxin substance [13-16], the mechanism of arsenic-induced neurotoxicity is not so clear. Chaudhuri et al. reported that in neonatal rat brain cells and human fetal brain cells, NO production was increased after arsenic treatment [17]. Ding et al. reported that arsenic induces nitric oxide generation through activation of inducible nitric oxide synthase (iNOS). Excessive production of nitric oxide has been implicated in neurodegenerative diseases [18]. It indicated that arsenic can induce overproduction of NO by inducing nitric oxide in the brain. A high concentration of NO reacts with oxyradical to product RNS including ONOO-, resulting in derangement of cell metabolism, breakage of DNA/RNA chains and tissue damage [19]. 8-Nitroguanine is a product of nucleic acid, which has been previously damaged by reactive nitrogen species resulting in further mutations and carcinogenesis [7,8,20]. In the present study, strong expression of 8-nitroguanine was shown in cerebral cortex and cerebella cortex of mice exposed to arsenic sub-chronically.

Our results showed that in the nucleus, where DNA resides, very intense 8-nitroguanine was present; whereas in the cytoplasm, the occupational location of RNA, a weak 8-nitroguanine signal was detected. Because 8-nitroguanine has a strong expression in neuron cells exposed to arsenic, it may be associated with DNA damage of nerve cells by arsenic. These results suggested that arsenic exposure may lead to nucleic acid damage in neuron cells. Moreover, 8-nitroguanine staining results demonstrated that 8-nitroguanine was presented much stronger in the nucleus than in the cytoplasm. 8-Nitroguanine exhibits rapid depurination in cells, and it is more stable in RNA than in DNA [21] indicating that strong expression of strong expression of 8-nitroguanine in arsenic exposure may be associated with both DNA/RNA damage in the neural cells. Our previous studies had reported that exposure to arsenic induced oxidative DNA damage of neurons in cerebral cortex [22]. These results implied that neurons of cerebral and cerebellum cortex may be the major target cells of arsenic neurotoxicity.

Taurine is the most abundant free amino acid in many tissues and has protection against adverse effect of ROS caused by various toxic substances [23-26]. The results showed relatively light pathological changes and weak expression of 8-nitroguanine in nervous cells of the mice administered both arsenic and taurine. It was indicated that administration of taurine can alleviate pathological changes and DNA damage caused by arsenic through the RNS signal pathway. The protections of taurine may be associated with the reduction of oxyradicals so as to prevent the reaction of NO with oxyradicals to overproduce ONOO^-^. Taurine has been shown to inhibit iNOS expression in phagocytes, resulting in suppressed nitric oxide production [27]. For example, arsenic-induced neuron damage is related to inflammation, NO, and apoptosis, and many factors (such as iNOS, ONOO^-^, etc) are related to the cause of DNA damage. In this way, we speculate that the correction of these molecules and morphological changes may lead to neurobehavioral improvement in patients, thus, the treatment using taurine may represent an ideal approach for improving function after chronic exposure to arsenate in drinking water. In order to clarify the functional role of the 8-nitroguanine, quantifiable method for various NO-mediated carcinogenesis models is under way in our laboratory.

## Conclusion

The most important findings of this study suggest that the neuroprotective effect of taurine against pathological changes and nucleic acid damage is due to reactive nitrogen species in cerebrum and cerebellum of mice exposed to arsenic. The effects are probably mediated by the inhibition of inflammatory responses (i.e., iNOS). Taurine itself possessed either free radical scavenging or antioxidative activity, whereas it may reduce superoxide anion formation probably through inhibiting neutrophil activation. We speculate that the correction of these molecules and morphological changes may lead to neurobehavioral improvement in patients, thus, the treatment using taurine may represent an ideal approach for improving function after chronic exposure to arsenate in drinking water.

## Competing interests

The authors declare that they have no competing interests.

## Authors’ contributions

NM carried out the antibody preparation and immunohistochemistry and drafted the manuscript. MS carried out the statistical analysis, participated in the immunohistochemical analysis. SK conceived of the study, and participated in its design. HS carried out the animal study and participated in tissue preparation. FP carried out the animal study, participated in tissue preparation, and participated in its design.
